# Supplementary figures and images for: Systematically improved in vitro culture conditions reveal new insights into the reproductive biology of the human parasite Schistosoma mansoni
Source: PLoS Biol. 2019 May 8;17(5):e3000254. doi: 10.1371/journal.pbio.3000254 (PMC6505934; doi:10.1371/journal.pbio.3000254)

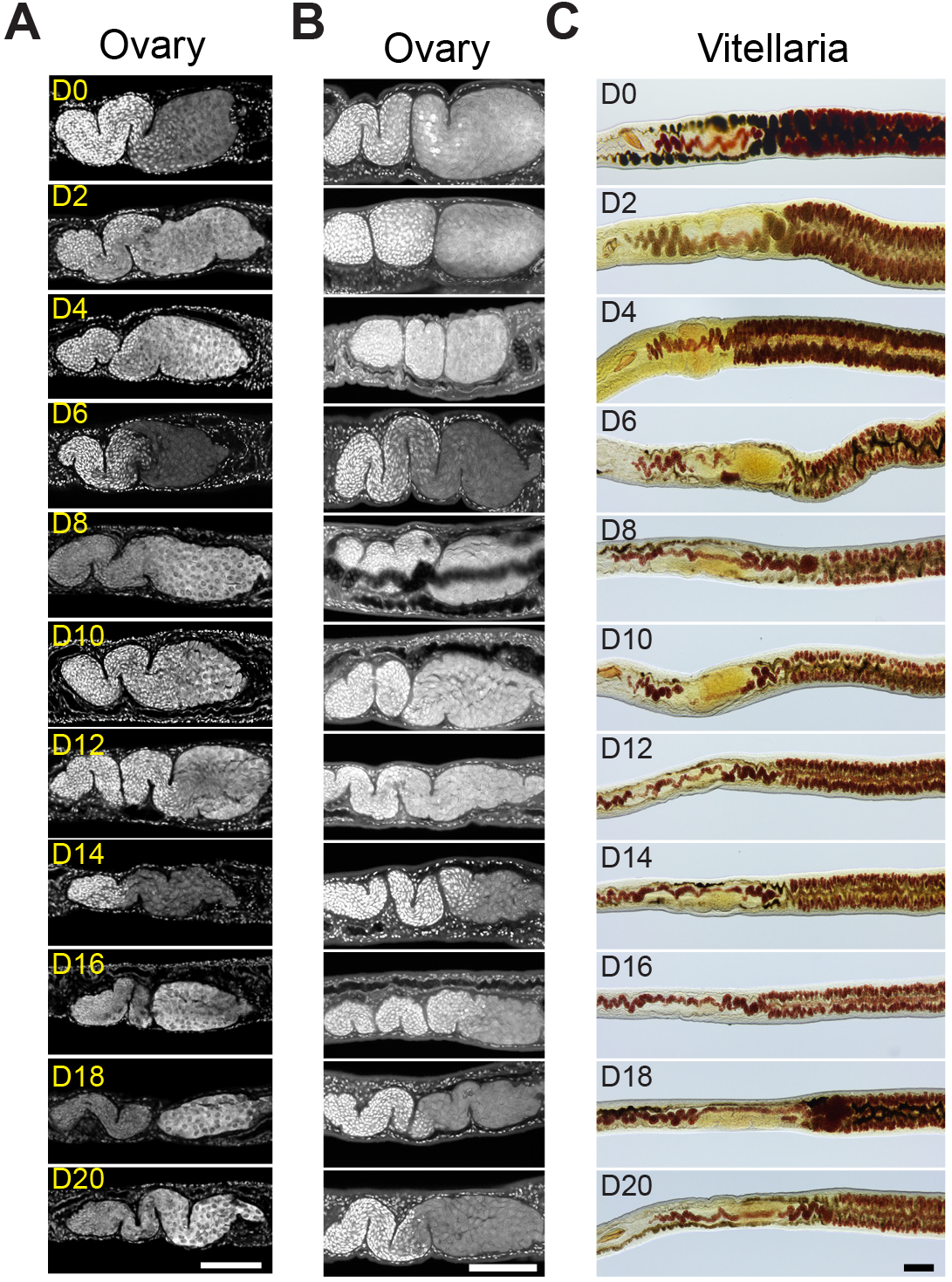

Supplement: S1 Fig — Ovaries of females in (A) BM169 or (B) ABC169 between D0 to D20 of in vitro culture labeled with DAPI. Differentiated oocytes are present in the posterior regions (right) of ovaries during in vitro culture regardless of culture condition. (C) Fast Blue BB labeling showing the maintenance of mature vitellocytes during culture in BM169. Representative images from 3 biological replicates with n > 10 parasites. Scale bars: 100 μm. ABC169, Ascorbic Acid, Blood Cells, Cholesterol, and BM169; BM169, Basch’s medium 169; D, day. (TIF) [file pbio.3000254.s001.tif]

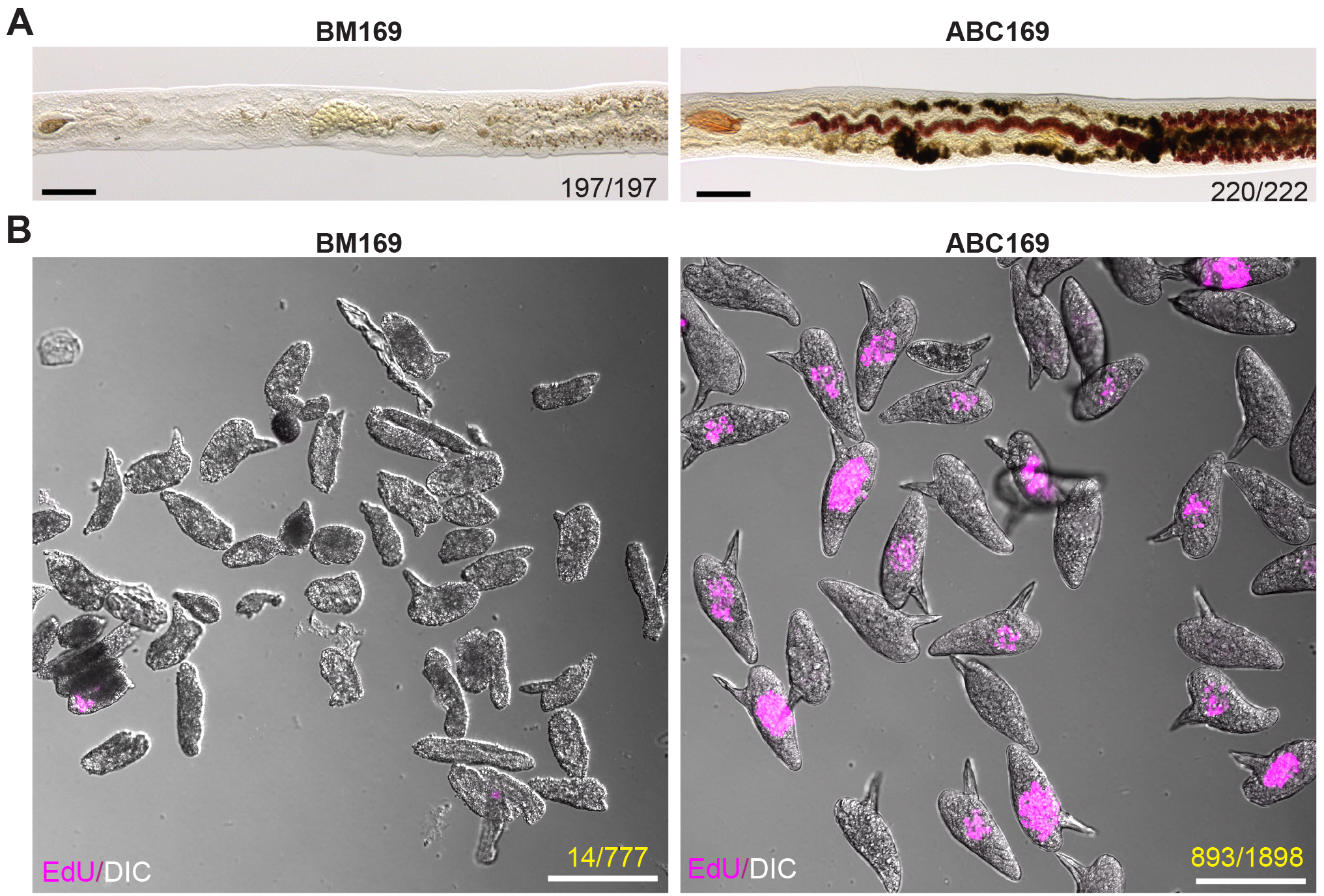

Supplement: S2 Fig — (A) Fast Blue BB labeling of paired adult female S. mansoni in BM169 or ABC169 at D30 of culture. Representative images from 3 separate experiments with n > 197 parasites. (B) EdU-labeled embryonic cells from eggs laid between D28 and D30 by paired adult females in BM169 or ABC169. Eggs laid by worms cultured in ABC169 appear normal in morphology, while those from BM169 were tiny and deformed. Representative images from 3 separate experiments; n-values indicate fraction of eggs that contain EdU+ proliferative embryonic cells. Scale bars: A, B, 100 μm. ABC169, Ascorbic Acid, Blood Cells, Cholesterol, and BM169; BM169, Basch’s medium 169; D, day; EdU, 5-ethynyl-2′-deoxyuridine. (TIF) [file pbio.3000254.s002.tif]

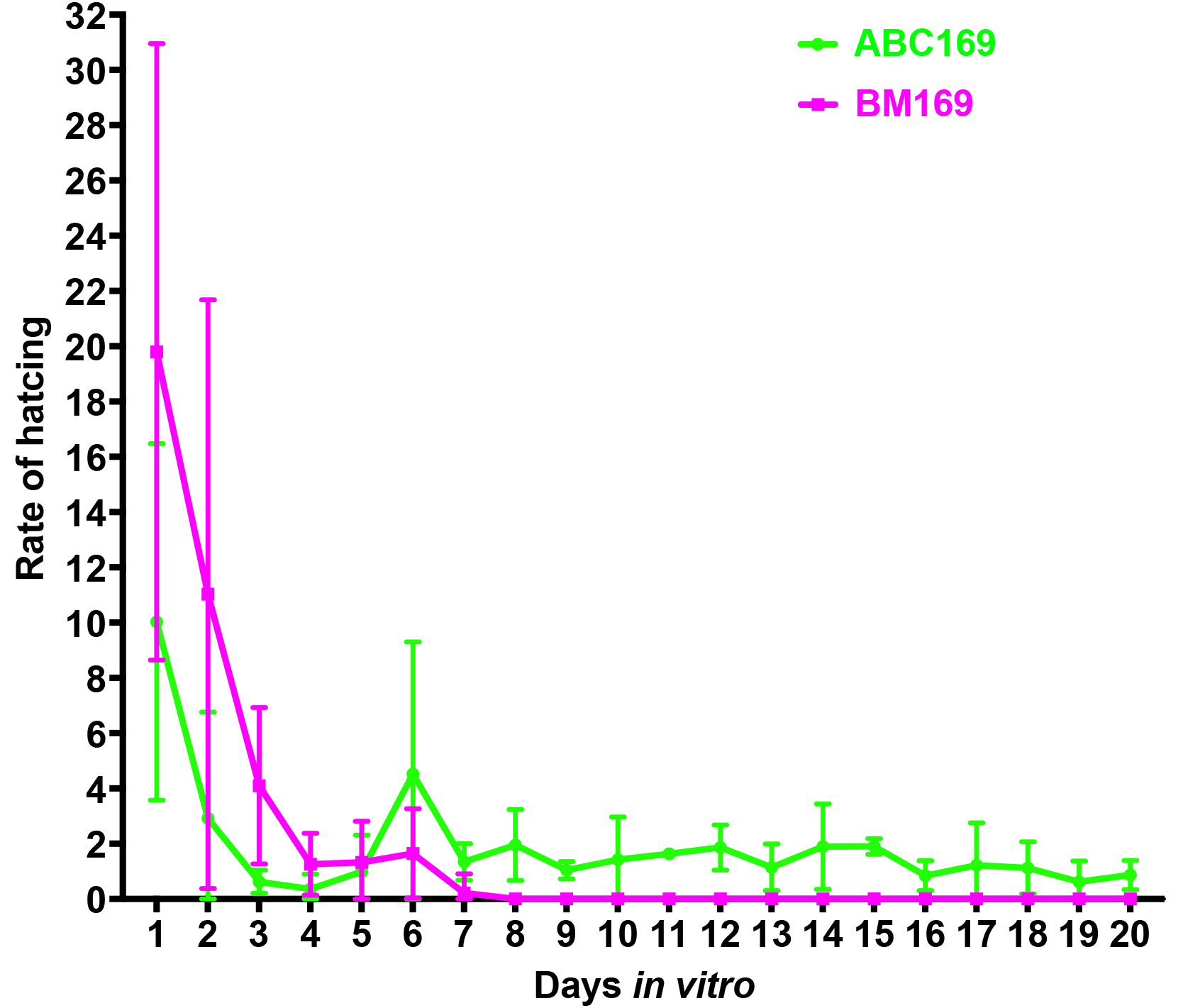

Supplement: S3 Fig — Data points represent mean values from 4 independent experiments. Error bars represent 95% confidence intervals. Underlying primary data can be found in S1 Data. ABC169, Ascorbic Acid, Blood Cells, Cholesterol, and BM169; BM169, Basch’s medium 169 (TIF) [file pbio.3000254.s003.tif]

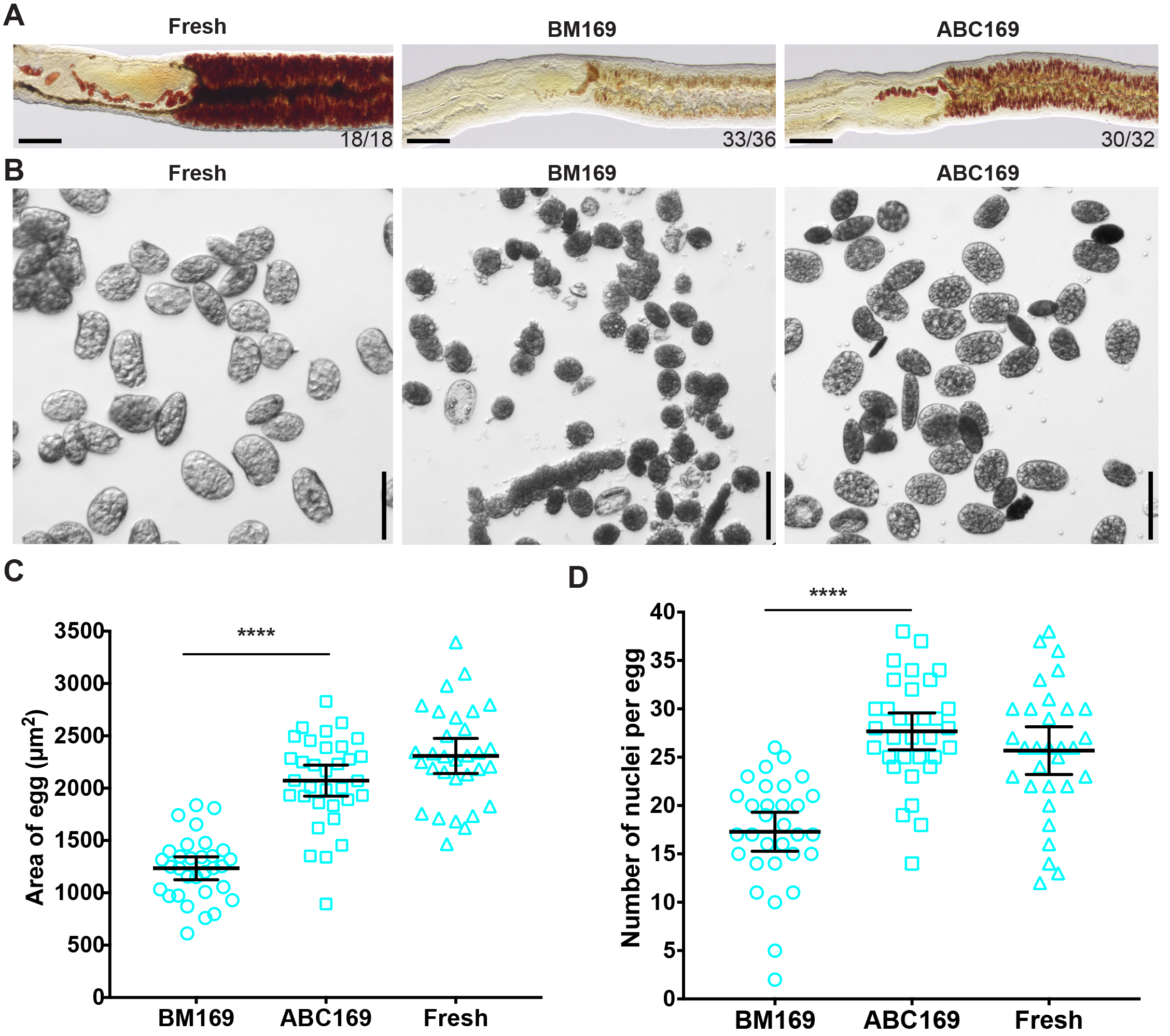

Supplement: S4 Fig — (A) Vitellaria visualized by Fast Blue BB staining in paired adult female S. japonicum in freshly perfused parasites (“Fresh”) or BM169/ABC169 at D15 of culture. Representative images from 3 separate experiments. (B) Morphology of eggs laid by freshly perfused female worms on first two days or paired adult female S. japonicum in BM169 or ABC169 on D15. Representative of 3 experiments. (C–D) Quantification of the (C) size, (D) number of DAPI-labeled nuclei from eggs laid by freshly perfused female worms (Fresh, n = 31 eggs) or laid by parasites cultured in BM169 (n = 31 eggs) or ABC169 (n = 33 eggs) on D15. ****p < 0.0001, t test. Error bars represent 95% confidence intervals. Underlying primary data for panels C–D can be found in S1 Data. Scale bars: A, B, 100 μm. ABC169, Ascorbic Acid, Blood Cells, Cholesterol, and BM169; BM169; Basch’s medium 169; D, day. (TIF) [file pbio.3000254.s004.tif]

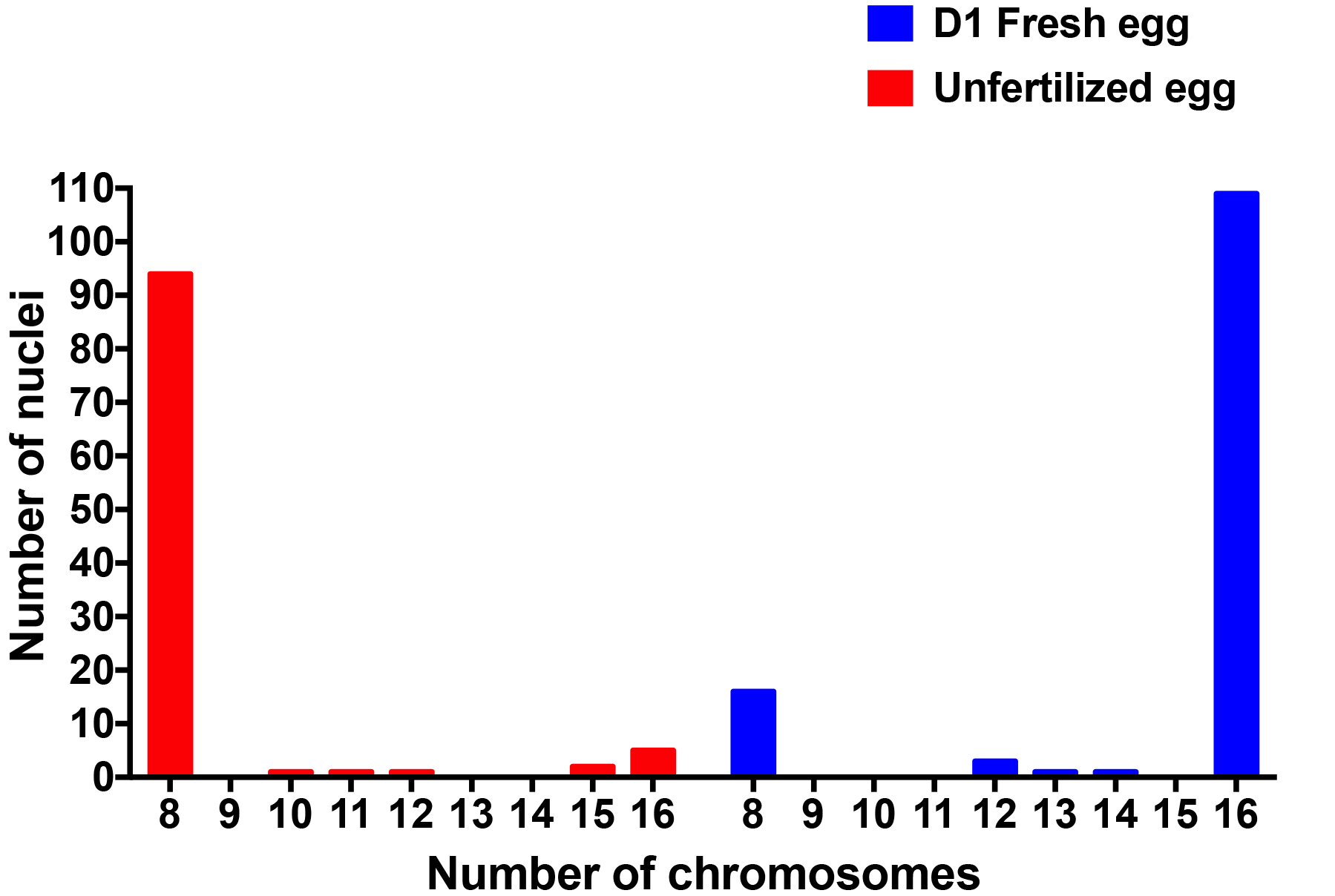

Supplement: S5 Fig — Plot showing number of chromosomes from karyotypes obtained from eggs laid by freshly perfused worm pairs on D1 of culture (“D1 Fresh eggs,” blue) or females paired with decapitated and castrated male segments (“unfertilized eggs,” red). Underlying primary data can be found in S1 Data. D, day. (TIF) [file pbio.3000254.s005.tif]

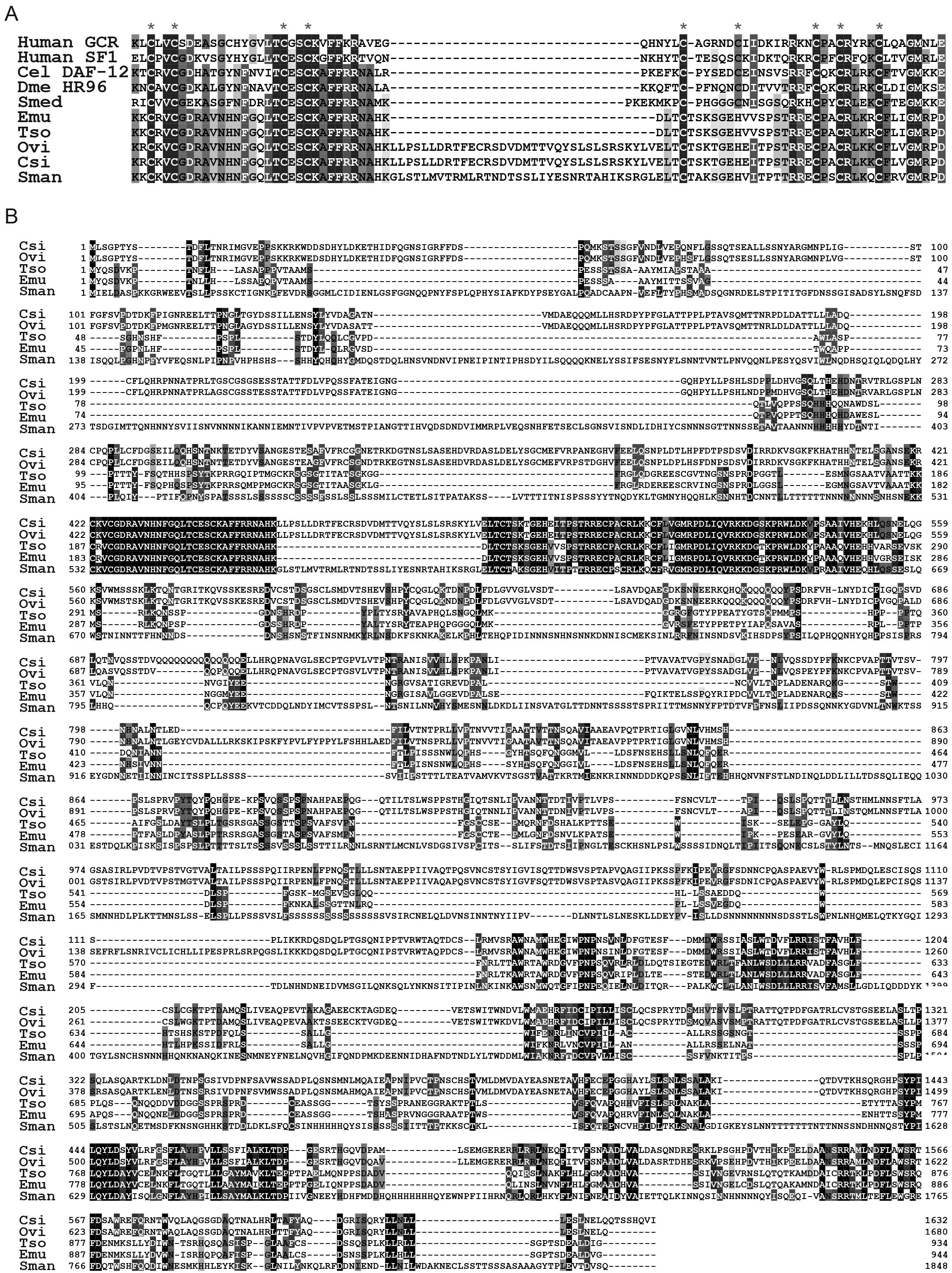

Supplement: S6 Fig — (A) Sequence alignment showing Smp_248100 shares a conserved DBD with other vertebrate and invertebrate NRs. Unlike the nonparasitic groups, parasitic flatworms shared a conserved histidine residue (indicted in yellow) in the position of the second conserved cystine (indicted in red) in the second zinc finger. (B) Full-length alignment showing C-terminus of Smp_248100 shares stretches of high amino-acid identity with orthologous proteins from other parasitic flatworms. Cel DAF-12, Caenorhabditis elegans nuclear hormone receptor family member daf-12 (NP_001041239); Csi, Clonorchis sinensis (csin106676); DBD, DNA-binding domain; Dme HR96, Drosophila melanogaster hormone receptor-like in 96 (NP_524493); Emu, Echinococcus multilocularis (EmuJ_001078800.1); Human GCR, Human glucocorticoid receptor (P04150); Human SF1, Human steroidogenic factor 1 (Q13285); NR, nuclear receptor; Ovi, Opisthorchis viverrini (T265_09674); Sman, Schistosoma mansoni (Smp_248100); Smed HR96, Schmidtea mediterranea (dd_Smed_v6_14067_0_3); Tso, Taenia solium (TsM_000102500) (TIF) [file pbio.3000254.s006.tif]

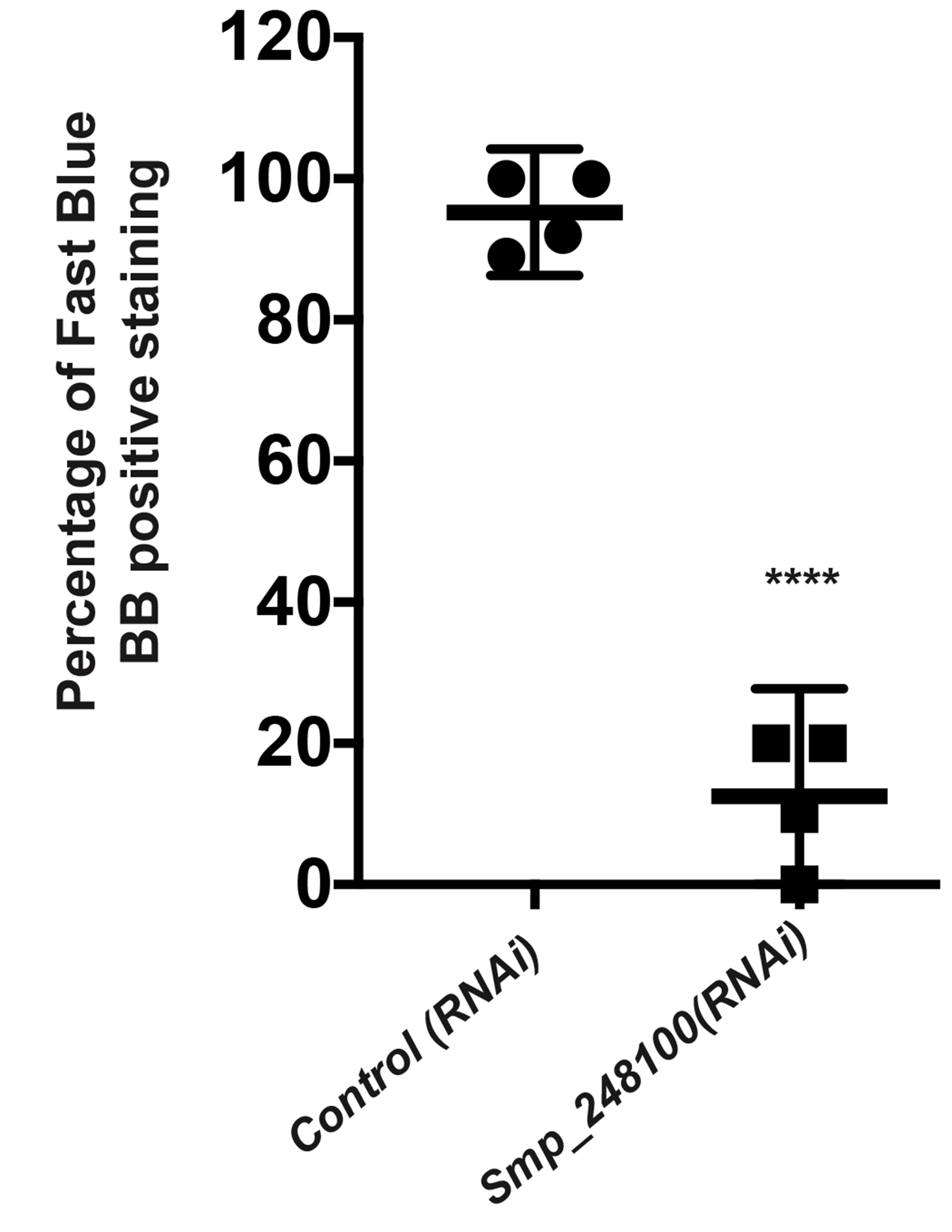

Supplement: S7 Fig — Plot showing percentage of Fast Blue BB positive labeling in Smp_248100 (RNAi) versus control (RNAi). Representative images from n > 39 parasites for each treatment examined in 4 separate experiments. ****p < 0.0001, t test. Error bars represent 95% confidence intervals. Underlying primary data can be found in S1 Data. D, day; RNAi, RNA interference. (TIF) [file pbio.3000254.s007.tif]
